# The Effect of a Working Memory Intervention Package on the Working Memory Performance of Primary School Students with Specific Learning Disabilities

**DOI:** 10.3390/jintelligence13020016

**Published:** 2025-01-27

**Authors:** Mehmet Okur, Veysel Aksoy

**Affiliations:** 1 Dede Korkut Faculty of Education, Kafkas University, Kars 36000, Türkiye; 2 Research Institute for Individuals with Disabilities, Anadolu University, Eskişehir 26000, Türkiye; vaksoy@anadolu.edu.tr

**Keywords:** working memory, specific learning disabilities, cognitive intervention, cognitive skills

## Abstract

This study examines the effects of a working memory (WM) intervention package on the WM performance of students with Specific Learning Disabilities (SLDs). A pre-test post-test experimental design was applied with 40 students, divided equally into experimental (20 students) and control groups (20 students). Data were collected using the Working Memory Scale (WMS), Raven’s Standard Progressive Matrices (RSPM), and the Working Memory Performance Tasks Form (WM-PTF). The experimental group demonstrated statistically significant improvements in WMS and WM-PTF scores relative to the control group (*p* < 0.006, *d* = 1.96 for WMS; *d* = 1.42 for WM-PTF). Additionally, a positive correlation was observed between the increase in WM performance and intelligence scores, suggesting that intelligence may influence WM gains. In conclusion, the WM intervention package was significant in improving the WM performance of students with SLDs, indicating that such interventions have significant potential for enhancing cognitive functions and memory. These findings highlight the critical role of WM interventions in contributing to the cognitive development of students with learning difficulties.

## 1. Working Memory and Cognitive Functions

Working memory (WM) is a cognitive mechanism that temporarily stores and processes information, serving as a foundation for various cognitive abilities. WM enables individuals to perform complex tasks, such as information processing, decision making, and problem solving. Research indicates that WM plays a central role in cognitive processes and significantly impacts cognitive success (Baddeley 2010). Multiple models have been proposed to explain the structure and function of WM, each offering different perspectives on its mechanisms, components, and interactions with cognitive skills. For example, Cowan’s (2005) Embedded Processes Model conceptualizes WM as a subset of long-term memory, limited to actively processed information facilitated by attention. Daneman and Carpenter’s (1980) Processing Efficiency Model suggests that WM’s capacity may vary according to the cognitive demands of tasks, where its efficiency is determined by processing effectiveness. Engle’s Executive Attention Model emphasizes the role of attention control, proposing that WM capacity is defined by individuals’ attentional control abilities. Ericsson and Kintsch’s (1995) Long-Term Working Memory Model highlights the dynamic interaction between WM and long-term memory, enabling rapid retrieval of information during complex tasks. Baddeley’s (2012) Multicomponent Model describes WM as a system of interconnected components—the central executive, the phonological loop, the visuospatial sketchpad, and the episodic buffer—working across different cognitive modalities. Finally, Dehn’s (2008) Integrated Working Memory Model argues that WM functions as a holistic mechanism, engaging both short-term and long-term memory to support multiple simultaneous information sources.

WM’s relationship with core cognitive skills, such as fluid intelligence, further underscores its importance. Fluid intelligence encompasses adaptive reasoning, problem solving, and abstract thinking—abilities that rely heavily on the efficient use of WM (Baddeley 2012). High WM capacity enables individuals to engage in more complex tasks, utilize attention effectively, and access information rapidly, highlighting WM’s essential role in cognitive and academic success. Research has demonstrated that WM capacity plays a critical role in fluid intelligence and also in general cognitive functions. The ability to process multiple pieces of information simultaneously significantly contributes to success in intelligence tests (Klingberg et al. 2005). In this context, it is crucial to distinguish between WM and short-term memory (STM). STM primarily refers to the temporary storage of information without manipulation, while WM includes both the storage and active manipulation of information (Baddeley 2010; Maehler et al. 2019). This distinction is important for both assessment and intervention purposes.

WM also plays a critical role in core cognitive functions, such as learning, attention, and executive processes. WM capacity determines how successful individuals will be in these cognitive functions. Engle (2002) emphasized that WM capacity is closely related to attention management and the inhibition of distractions. Individuals with limited WM capacity tend to commit more errors, particularly in tasks that demand sustained attention. This can negatively impact their performance in both academic and daily life (Gathercole and Alloway 2008). WM capacity has practical implications for educational settings, as it influences how students follow instructions, solve problems, and engage in learning activities (Holmes et al. 2009). In learning processes, the limited capacity of WM makes encoding information and transferring it to long-term memory more challenging. Therefore, strengthening WM can have a significant impact on students’ academic success (Alloway 2010).

### 1.1. Memory Difficulties and Working Memory in Specific Learning Disabilities

SLD is defined as neurodevelopmental disorders in which individuals experience difficulties in learning processes, negatively impacting their academic performance (American Psychiatric Association 2013). Despite having normal or above-normal intelligence levels, these individuals face significant challenges in areas like reading (dyslexia), writing (dysgraphia), or mathematics (dyscalculia). This study aims to provide a more comprehensive understanding of how WM interventions can be tailored to meet the unique cognitive profiles of students with SLD, thereby contributing to both theoretical insights and practical applications in educational settings. Understanding the memory difficulties observed in these students is crucial for comprehending SLD and developing effective interventions (Gathercole and Alloway 2008). The existing literature suggests a possible link between WM and SLD, implying that challenges in these two areas may influence each other (Gathercole and Alloway 2008). The mutual impact of these difficulties highlights the importance of interventions that consider this relationship and address specific needs (Maehler and Schuchardt 2016; Peng et al. 2018).

Research indicates that WM is a significant factor in primary cognitive difficulties in SLD (Gathercole and Alloway 2008; Swanson 2011). Students with SLD exhibit weaknesses in various WM subcomponents (e.g., verbal memory and visuospatial memory). In particular, the phonological loop is a critical factor that may contribute to their difficulties in tasks like reading and writing (Gathercole and Pickering 2010). Difficulties in the phonological loop lead to deficits in processing and temporarily storing verbal information, negatively affecting reading and language development (Swanson and Siegel 2011). The theoretical framework for this study is anchored in the WM model proposed by Baddeley (2010), which includes the central executive, the phonological loop, and the visuospatial sketchpad as key components for information storage and manipulation. While this study is attached to Baddeley’s (2010) multi-component model of WM, alternative models, such as Cowan’s (2005) embedded-processes model and Dehn’s (2015) comprehensive framework, offer additional insights into how WM functions within different cognitive and educational contexts. Understanding these theoretical underpinnings is essential for developing effective intervention strategies that address the specific WM deficits seen in students with SLD.

Another critical aspect of WM challenges in SLD involves difficulties in processing and recalling spatial information. This can result in failures, especially in mathematical problem solving and geometry (Passolunghi and Mammarella 2012). The components of WM not only affect students’ academic performance but also influence their daily cognitive functioning (Peng and Fuchs 2016). Low performance in WM diminishes the capacity to handle information efficiently and obstructs its transfer to long-term memory, making it challenging for information to be retained permanently. These deficits can contribute significantly to academic difficulties. For instance, a study investigating the impact of WM on academic performance demonstrated that WM serves as a stronger indicator of educational success compared to intelligence (Alloway and Alloway 2010).

In summary, there is a reciprocal relationship between WM and SLD, with each influencing the other. Given that WM is a critical factor affecting the cognitive functions of individuals with SLD, interventions aimed at improving WM performance are crucial for addressing the challenges faced by this population (Holmes and Gathercole 2014). Research suggests that targeted, evidence-based WM interventions can enhance not only academic outcomes but also broader cognitive skills, facilitating better learning strategies and adaptive functioning (Hu and Hu 2023). Therefore, implementing structured WM training programs in educational settings is essential for supporting the cognitive development of students with SLD.

### 1.2. Working Memory Interventions: Strategies and Effectiveness

Interventions aimed at enhancing WM performance are critical, as they play a key role in supporting students’ learning processes. This study situates itself within the broader framework of interventions that aim to enhance WM performance specifically for students with SLD, focusing on immediate improvements in WM capacity. These interventions are structured around strategies that help individuals optimize their memory capacity, enabling more effective information processing (Gathercole and Alloway 2008). Such cognitive strategies are reported to facilitate learning and recall processes (Baddeley 2010). Notably, interventions designed to strengthen WM in students with SLD have demonstrated significant positive effects on WM-related tasks (Swanson 2011). While WM training programs have consistently demonstrated effectiveness in enhancing WM and related cognitive functions, their impact on broader academic achievement remains limited and widely debated. The literature indicates that these programs, including those aimed at students with SLD, primarily yield improvements in WM itself. Meta-analyses, such as that by Melby-Lervåg and Hulme (2013), highlight a substantial body of evidence suggesting that, despite WM gains, significant enhancements in academic skills are generally absent or minimal. In a study by Melby-Lervåg and Hulme (2013), WM training was reported to provide short-term improvements in specific tasks; however, it showed no substantial effect on broader cognitive abilities. Another study found that children with SLD displayed some short-term improvements following WM training, although similar outcomes were observed in the control group, indicating that the effect of the training was minimal (Spencer-Smith et al. 2020).

Recent studies, such as that by Maehler et al. (2019), provide further insight into the nuanced impact of WM training on cognitive processes, emphasizing that while cognitive gains are observed, their transferability to long-term academic outcomes is limited. This emphasizes the importance of evaluating not only immediate cognitive improvements but also the sustainability and educational relevance of these effects over time.

Increasingly, recent research has focused on WM interventions incorporating technology-enhanced and adaptive learning approaches, yet the evidence still suggests that such advancements do not consistently translate into broader academic achievement gains (Hu and Hu 2023).

By incorporating rehearsal, chunking, and visualization as primary methods, this study builds on established techniques but seeks to measure their comprehensive impact on WM performance. These strategies were chosen specifically because they address common deficits seen in students with SLD, such as difficulties in retaining and processing verbal and visual information. Rehearsal helps reinforce memory retention through repetition, which is particularly beneficial for students who struggle with transferring information to long-term memory (Gathercole and Alloway 2008; Titz and Karbach 2014). Chunking breaks down complex information into manageable units, facilitating the recall of sequences and structured data, which supports students who have trouble processing large amounts of information at once (Cowan 2005; Chen and Cowan 2009). Visualization aids in creating mental images that anchor abstract concepts to tangible representations, benefiting students with weaknesses in visuospatial memory by making information more concrete and memorable. These strategies were selected due to their effectiveness and adaptability in addressing the unique cognitive challenges experienced by students with SLD, as supported by recent findings in educational psychology (Zaidi and Zaidi 2022; Wang and Chung 2021). This selection aligns with evidence indicating that visual and auditory strategies, along with chunking strategies that reduce cognitive load, strengthen multi-sensory approaches by supporting both visual and auditory learning styles (Mayer and Moreno 2003).

It should be noted that this study does not include data collection on the long-term effects of WM interventions or their broader impact on cognitive skills. The primary focus is on the immediate outcomes of these strategies and their short-term implications for WM performance in students with SLD. The results of intervention research clearly demonstrate the favorable effects of WM interventions on students with SLD. The meta-analysis by Rao et al. (2023) highlighted significant improvements in the WM performance of students with SLD, emphasizing the positive impact of these interventions on WM outcomes. Similarly, Peijnenborgh et al. (2016) reported in their meta-analysis that substantial improvements were observed in both verbal WM and visuospatial WM following intervention. These meta-analyses consistently show positive contributions to the WM performance of individuals with SLD.

Holmes and Gathercole (2014) found that WM interventions improved students’ academic achievement and cognitive skills. Likewise, Alloway and Alloway (2010) noted that such interventions enhance verbal and visual memory performance, contributing to learning processes. Another area where WM interventions have proven effective is in executive functions; enhancing these functions helps students succeed in problem solving, planning, and attentional tasks (Swanson and Siegel 2011). Researchers, such as Gathercole and Alloway (2008), emphasize that WM interventions lead to improvements, particularly in verbal and visuospatial memory components.

Many of the studies in the literature involving individuals with SLD show that WM interventions are often computer-based (Horowitz-Kraus and Breznitz 2009; Shiran and Breznitz 2011; Fälth and Brkovic 2021) and applied individually (Banales et al. 2015; Fälth and Brkovic 2021). Computer-based WM interventions have been shown to be particularly effective due to their adaptability and interactive nature, which can cater to individual student needs (Fälth and Brkovic 2021). WM intervention studies include both individuals with SLD (Peng and Fuchs 2016; Al-Zoubi and Rahman 2015; Novaes et al. 2019; BahrAsemani et al. 2021) and individuals with both SLD and ADHD (Gray et al. 2012; Gropper et al. 2014; Heishman 2015). These studies generally report positive outcomes. Few studies include strategy instruction (Peng and Fuchs 2016; Novaes et al. 2019). However, given the well-known difficulties and deficiencies that students with SLD face in generating strategies (Pintrich et al. 1994; Gautam 2023), there is a need for more interventions, such as direct instruction, that incorporate modeling and hands-on strategy instruction (Stockard et al. 2018; Adams and Engelmann 1996). Future research should focus on hybrid approaches that combine computer-based interventions with strategy instruction to maximize both engagement and effectiveness in SLD populations.

### 1.3. Aims and Rationale

The primary aim of this study is to develop a comprehensive WM intervention package tailored to primary school students with SLD and to evaluate its immediate impact on their WM performance. Specifically, the study aims to assess changes in the visual and verbal memory subdomains between the experimental and control groups and measure overall improvements in WM performance.

This study offers significant contributions to the literature and distinguishes itself from previous research in several ways. First, the WM intervention is designed to be delivered via remote and group-based instruction, increasing accessibility and feasibility for students with SLD. Additionally, this intervention employs a structured instructional approach, following the modeling, guided practice, and independent practice steps of direct instruction. In addition to the three primary instructional strategies supporting WM, internal repetition, visualization, and chunking techniques are also incorporated. In the literature, these types of applications are generally presented as isolated tasks, individual technology-based applications, or single activities, whereas this study presents them within a comprehensive strategy instruction framework. In this sense, the intervention package is structured as an integrated WM model (Dehn 2015) addressing a gap in existing research by providing a more concrete and applicable framework to translate WM interventions into an educational support format. This integrated approach aims to make the abstract concept of WM more tangible and applicable for students and educators, thereby enhancing its practical utility in school settings. Furthermore, this study uniquely examines the relationship between WM intervention effectiveness and students’ intelligence scores, providing insights into how intelligence may moderate the benefits of WM interventions for children with SLD. By including a focus on fostering awareness of both WM and memory within the intervention content, this study aims to deepen understanding of these cognitive functions. Given these distinctive features, this research is expected to make a valuable contribution to the literature, particularly in terms of developing more practical and accessible WM interventions for students with SLD.

The study is designed to test the following hypotheses:

**H1.**  *The WM intervention package will result in statistically significant improvements in the visual memory subdomain scores of students in the experimental group compared to the control group.*

**H2.**  *The WM intervention package will lead to statistically significant improvements in the verbal memory subdomain scores of students in the experimental group compared to the control group.*

**H3.**  *The total WMS scores of students in the experimental group will show statistically significant increases compared to those in the control group following the WM intervention.*

**H4.**  *The WM intervention package will result in statistically significant improvements in the total WM-PTF scores of students in the experimental group compared to the control group.*

**H5.**  *There will be a statistically significant positive correlation between the changes in pre-test and post-test WMS scores and the general intelligence test scores of students in the experimental group after the WM intervention.*

Although H3 could be perceived as the combined outcome of H1 and H2, it has been formulated as an independent hypothesis to explore overall improvements in working memory performance. This is because different combinations of outcomes are possible (e.g., H1 supported while H2 is not, or vice versa), and examining total WMS scores separately allows for a more comprehensive understanding of the intervention’s effects.

## 2. Methods

### 2.1. Research Design

This study employed a pre-test post-test experimental design, incorporating a control group, to assess the impact of the WM intervention package on the WM performance of students with an identified SLD. The design is a thorough method frequently used to examine the influence of an independent variable on a dependent one (Babbie 2010). In this context, the dependent variable refers to the WM performance of students with SLD, while the independent variable is the group assignment (intervention and control groups). The experimental group was subjected to the targeted intervention program aimed at improving WM performance, while the control group did not receive any form of intervention, allowing for a direct assessment of the intervention’s effectiveness by comparing the outcomes between the two groups.

The sample included forty primary school students diagnosed with SLD selected using purposive sampling to ensure that participants met specific inclusion criteria. This method was chosen to target students who would benefit most from the WM intervention. Detailed participant characteristics and selection criteria are described in the “Participants” section. However, after the post-test, the control group was offered recommendations for interventions and resources, including books and applications related to working memory.

### 2.2. Study Group and Group Assignment

The participants include students diagnosed with SLD who are receiving education at special education and rehabilitation centers in Turkey. Criterion sampling was used to select the study group. The participant selection criteria are as follows:

1. According to the Special Needs Report for Children (SNRC), a diagnosis of SLD was obtained from state hospitals.
2. Students must be between the ages of 9 and 10.
3. Families and students must voluntarily agree to participate in the research.
4. Students must score within the normal or above-normal range on an intelligence test.
5. Students must have access to a computer at home and be in an environment suitable for participation in the study.

For the group assignment process, the participants were randomly assigned to either the experimental or control group using a transparent lottery method to ensure unbiased distribution. The process involved the following steps:

1. Preparation of Student Lists: Rehabilitation centers with a higher number of students diagnosed with Specific Learning Disabilities (SLDs) were contacted, and a list of eligible students who met the inclusion criteria was prepared.
2. Information and Consent Process: Both the institution administrators and the parents of the students were informed about the study, and written informed consent forms were obtained. Participation in the study was entirely voluntary.
3. Randomization Using a Lottery Method: The names of all eligible participants were written on individual slips of paper and placed into a container. Names were then drawn one at a time, with each selected student alternately assigned to the experimental or control group.

This method was chosen to ensure transparency and fairness in the assignment process while eliminating selection bias. By relying entirely on chance, the process safeguarded the internal validity of the study, ensuring that any observed differences in outcomes could be attributed to the intervention rather than pre-existing differences between the groups.

The participants included students diagnosed with SLD who were receiving education at special education and rehabilitation centers in Turkey. In Turkey, the diagnosis of SLD is determined based on DSM-V criteria, as specified by the Ministry of Health. This diagnosis is officially established by a clinical team composed of child psychiatrists and pediatricians. SLD is categorized under a general label rather than being divided into subtypes, such as dyslexia or dyscalculia. Due to the frequent overlap between subtypes (e.g., individuals with dyslexia often experience writing difficulties) and the rarity of cases with isolated subtypes, this study focuses on the general SLD diagnosis (Büber et al. 2020). The age range of 9–10 years was chosen because it represents a critical developmental period for the effectiveness of WM interventions. WM skills are actively developing during this stage, and this period aligns with an educational phase where foundational academic skills, such as reading and mathematics, are reinforced. These skills are frequently impacted by SLD. Additionally, the proximity of this age group to the onset of abstract thinking supports the effectiveness of foundational strategy instruction (Melby-Lervåg and Hulme, 2013; Dehn 2015).

Forty students who met the inclusion criteria, including age, diagnosis, volunteering, RSPM, and WMS, were randomly assigned to either the experimental or the control group. A lottery method was used to ensure unbiased and transparent distribution. This randomization process minimized selection bias and safeguarded the internal validity of the study, ensuring that any observed differences in outcomes could be attributed to the intervention rather than pre-existing differences between the groups. The descriptive characteristics of the participants, as well as the WMS and RSPM test averages, are provided in Table 1.

RSPM scores between the experimental and control groups showed no significant difference (Mann–Whitney *U* = 201.50, *p* = 0.97), indicating comparable cognitive performance. Similarly, IQ scores between the groups also showed no significant difference (Mann–Whitney *U* = 206.5, *p* = 0.86). This test was chosen due to the non-parametric nature of the RSPM and IQ data distribution. These results indicate that both groups’ initial fluid intelligence performance was comparable. The mean ages of both groups in the study were very similar.

The WMS pre-test results were examined to assess whether a significant difference existed between the experimental and control groups. The Shapiro–Wilk test was applied to assess the normality of the distribution for the WMS pre-test total score. The Shapiro–Wilk test was chosen due to its robustness for small sample sizes. The results (*W* = 0.97; *p* = 0.47) show that the *p*-value exceeds the 0.05 threshold, indicating normal distribution of the data. Accordingly, an independent samples t-test, a parametric method, was utilized to compare the mean scores between the two groups. An independent samples *t*-test was used as the data met the normality assumption, making it an appropriate parametric method for comparing group means. The analysis indicated no significant differences in WMS pre-test scores between the experimental and control groups (*t* = −0.69; *p* > 0.05). These findings suggest that both groups were equivalent in their intelligence scores and WMS performance at the start, thereby ensuring that any subsequent differences observed could be attributed to the intervention. Although the randomization process resulted in an unequal distribution of grade levels between the experimental and control groups, there were no significant differences in age, WMS score, or IQ (*p* > 0.05). Therefore, grade level was not considered a confounding factor in this study.

### 2.3. Dependent Variable and Measurement Instrument

In this study, the WMS and the WM-PTF were used as dependent variables to observe changes in the WM performance of students with SLD. The independent variable of the study is the WM intervention package. The subdomains of WM performance were also included as additional dependent variables. The data collection instruments for all dependent variables in the study are listed below.

Working Memory Scale (WMS): The WMS was developed to assess the WM performance of children aged 5–10 (Ergül et al. 2018). The scale consists of nine subtests across four dimensions: verbal/visual STM and verbal/visual WM. The verbal STM subtests include digit and word recall, while the visual STM subtests involve pattern and block recall tasks. In the verbal WM dimension, tasks include backward digit recall and first word recall, whereas the visual WM dimension involves spatial recall and selecting the different item tasks. Each test is administered with two trials of increasing difficulty, and scoring is based on the number of correct answers. The WMS has demonstrated strong psychometric properties. Validity analyses have indicated robust content validity, as the scale was evaluated by experts and deemed appropriate for children aged 5–10. Construct validity was supported by KMO values ranging from 0.64 to 0.82, confirming its suitability for factor analysis, and statistically significant Bartlett’s test results (*p* < 0.05). The explained variance for subscales ranged from 58% to 72%, indicating a well-defined factor structure. In terms of reliability, the WMS showed high internal consistency, with Cronbach’s alpha coefficients between 0.98 and 0.99 across subscales, signifying excellent reliability. Test–retest reliability was confirmed through Pearson correlation coefficients, demonstrating consistent results over time. Item–total correlations ranged from 0.40 to 0.94, indicating strong item discrimination and internal reliability. The WMS was included as a measurement tool independent of the intervention materials to evaluate post-intervention working memory performance. This ensured a more unbiased assessment of the intervention’s effects.

Raven’s Standard Progressive Matrices (RSPM) Test: The test is a culturally neutral intelligence test that measures general intelligence (the ‘g’ factor) and abstract reasoning abilities in children aged 7–15 (Raven et al. 2004). It has been widely used across various countries and is independent of cultural differences. In Turkey, it was standardized for individuals aged 7–15 by Sahin and Düzen (1994). The test assesses abstract thinking and reasoning abilities and, due to its non-verbal nature, is minimally influenced by factors like age, language, and sociocultural background. The RSPM consists of five sub-sections and a total of 60 items, in which individuals are asked to identify the missing part of a pattern from a set of images. The results are evaluated using percentile ranks based on chronological age (Raven 2003). The RSPM has demonstrated robust psychometric properties in terms of reliability and validity. Reliability analyses using the Two-Parameter Logistic (2PL) model reported an average item discrimination parameter (α) of 1.66, indicating high internal consistency, with an overall test reliability of approximately *r* ≈ 0.97. In the One-Parameter Logistic (1PL) model, reliability was slightly lower, with a fixed item discrimination coefficient of 1.41 for all items. Validity studies indicate that the RSPM has strong construct validity, remaining unaffected by demographic factors, such as age and gender. Both 1PL and 2PL models confirmed the one-dimensionality assumption, showing that the test effectively measures a single cognitive construct. These findings validate the strong psychometric quality of the RSPM and its sensitivity in accurately assessing abilities across a range of intelligence levels, particularly within the IQ range of 75–110 (Van der Elst et al. 2013).

Working Memory Performance Tasks Form (WM-PTF): The WM Performance Tasks Form (WM-PTF) was developed by the researcher to assess the subcomponents of working memory. Each subcomponent consists of 6 tasks, which are ordered from easy to difficult. These tasks include Visual–Spatial STM, Auditory STM, Visual–Spatial WM, Verbal WM, and Executive WM tasks. The prepared tasks were evaluated for content validity by three subject matter experts and then selected. Subsequently, decisions were made regarding how students should respond. The students’ responses were documented. Subsequently, decisions were made regarding how students should respond to the tasks. In this process, a numerical encoding response technique was used to enhance the processing capacity of all tasks. This technique required students to respond to the tasks using numerical codes. The numerical encoding response technique combined recall and processing demands, transforming each task into a format that required more intensive processing. The maximum achievable score for these performance tasks is 30. The total application time ranges from 5 to 8 min. Psychometric analyses indicate that the WM-PTF demonstrates strong reliability and validity. Expert evaluations yielded a Content Validity Index (CVI) of 0.90, confirming content validity. Construct validity was supported by high item–total correlations, particularly for Phonological STM (r = 0.72), Verbal Total (r = 0.83), and Executive WM (r = 0.71), indicating alignment with the intended constructs. The overall Cronbach’s alpha was 0.81, with subtest reliabilities of 0.76 for Visual and 0.83 for Verbal tasks, demonstrating high internal consistency. These findings establish the WM-PTF as a valid and reliable tool for assessing working memory components in students with SLD. The WM-PTF assessment tool was developed independently of the intervention materials and strategies to ensure objective measurement and unbiased evaluation of the intervention’s effectiveness.

The reason for using the WM-PFT format in addition to the WM Test is to highlight the differences in their approaches to evaluating working memory processes. The WMS is based on Baddeley’s (2010) theory and employs a process model focused on STM and WM tasks. In contrast, the WM-PFT is grounded in Dehn’s (2008) Comprehensive Working Memory model, aiming to provide a more comprehensive evaluation by incorporating both recall and active processing of information. The WM-PFT differentiates itself from the WMS by integrating more processing methods into all tasks. This method ensures that tasks require not only retaining information but also focusing more intensively on active processing. By embedding processing demands into all tasks, the WM-PFT introduces a higher cognitive load for completing and responding to its tasks. On the other hand, the WM Test does not include subtests targeting executive working memory. These distinctions clearly differentiate the methods these two tools use to evaluate working memory performance.

### 2.4. Process

This section discusses the development process, content, objectives, and strategies of the WM intervention package. The stages of creating the package, the activities, and the goals are introduced, followed by a detailed explanation of the implementation steps.

#### 2.4.1. Development of the Working Memory Intervention

The Working Memory Intervention Package (WMIP) was developed and implemented by the researchers. The intervention was designed based on Dehn’s Comprehensive Working Memory Model (Dehn 2008), focusing on the components of STM, WM, and executive WM. Additionally, insights from studies by Dehn (2008), Swanson (2011), and Fälth et al. (2015) were integrated into the package. Activities were structured to target specific working memory components in alignment with the measured outcomes. The intervention was designed to incorporate technology-based applications and was made accessible online through the Zoom platform. Activities were organized into easy, medium, and difficult levels, enabling students to progressively learn and apply working memory strategies. Visual aids were used to engage primary school students, while verbal and auditory stimuli were reinforced with concrete words (Mayer and Moreno 2003; Paas et al. 2003).

Three special education experts contributed to the intervention’s development, and the content was refined through online meetings based on their feedback. The most effective activities were selected, and necessary revisions were made accordingly. Prior to the main implementation, a pilot study was conducted with four students to evaluate the clarity and applicability of the activities. Feedback from the pilot study informed further revisions to enhance task comprehensibility and interaction quality. To ensure the consistency of the intervention, each session was recorded via Zoom and subsequently reviewed by an independent observer using a protocol checklist. This process helped verify the reliability of the intervention and confirmed that the sessions were conducted as planned.

#### 2.4.2. Working Memory Intervention Package

The intervention package is a 5-week program consisting of a total of 45 sessions designed to develop WM skills systematically. This intervention program includes components targeting visual–spatial short-term memory and working memory, verbal short-term memory and working memory, and executive working memory. These components have been integrated into the 5-week intervention package. The program provides detailed strategies aimed at enhancing working memory performance, along with instructional methods for teaching these strategies. In the first week, sessions focused on familiarizing students with the intervention, introducing the concepts of memory and WM, and establishing effective online learning practices. During this period, students were introduced to each other and familiarized with the package through basic practice examples, enhancing their understanding of memory processes and WM skills in an online setting. Memory strategies—specifically, rehearsal, chunking, and visualization—were taught and practiced throughout the program. In the subsequent weeks, phonological and visual–spatial STM activities were implemented, allowing students to practice and develop their skills in using these strategies within structured tasks. This phase aimed to build foundational skills in strategy application, focusing on enhancing students’ ability to retain and recall verbal and visual–spatial information effectively.

As students progressed through the program, activities targeting verbal STM and WM, visual–spatial STM and WM, and executive WM were introduced to enhance fluency and automaticity in strategy use. These activities allowed students to apply memory strategies more fluidly in both phonological and visual–spatial contexts, bridging the gap between STM practice and working memory application. In the final phase, the program emphasized independent strategy application. Executive WM tasks were introduced to encourage students to apply learned strategies autonomously, reinforcing both WM capacity and strategic flexibility. Additional strategy instruction was provided to support students’ ability to self-regulate their memory strategies, promoting long-term retention and independent use of WM strategies in everyday tasks. Each week progresses according to specific goals and learning outcomes, as shown in Table 2.

The implementation of the intervention package was carried out by the first researcher. Prior to the intervention, both families and students were thoroughly briefed via the Zoom platform, and a tutorial video was shared to ensure clarity regarding the intervention’s objectives and procedures. A pilot study was conducted to fine-tune the process. The intervention program spanned three days per week over five weeks, with three sessions conducted each day, each lasting approximately 25–30 min, totaling 20 h of instruction. Sessions were held on Zoom, where clear objectives were presented at the beginning of each session to capture students’ attention and set expectations.

The direct instruction model guided the teaching process, where memory strategies were systematically introduced. Students were encouraged to articulate and explain the strategies they learned to reinforce understanding. During independent practice, students submitted their responses through the Zoom platform, where the researcher provided specific, formative feedback to support their progress. At the end of each session, strategies were reviewed to reinforce retention, and the session was evaluated to monitor student engagement and comprehension. To ensure the fidelity of the intervention, sessions were recorded and subsequently reviewed by an independent observer using a protocol checklist. This fidelity monitoring aimed to verify that the intervention adhered to the planned procedures and provided a reliable structure for future implementation. Upon completion of the intervention and post-tests, the control group received a two-week accelerated education program, including strategy training, while families were provided with materials and resources to support students’ learning and application of memory strategies, as well as information about WM interventions.

#### 2.4.3. Data Collection and Analysis

Data collection was conducted on an individual basis using RSPM, WMS, and WM-PTF. The assessments took place in a quiet environment to minimize distractions and improve concentration. The researcher provided students with a brief orientation regarding the assessment process to ensure that each participant felt comfortable and understood the procedure. Following the pre-test, a small gift (a 3D metal puzzle) was provided to each student as a token of appreciation to maintain motivation. Upon completion of the intervention package in the experimental group, post-test data were gathered from both the experimental and control groups. Students were allowed to select their own gifts following the post-test, which served as a further motivational incentive. To assess the normality of the pre-test and post-test results from the WMS and WM-PTF, skewness and kurtosis coefficients were calculated. Additionally, a Shapiro–Wilk test was conducted, with the results summarized in Table 3, to provide a comprehensive examination of data normality, ensuring the accuracy and robustness of subsequent statistical analyses.

According to the literature, skewness and kurtosis values between +2.0 and −2.0 are generally accepted as indicators of normal distribution (George and Mallery 2010; Kline 2023). For this study, skewness and kurtosis values for the WMS and WM-PTF tests, as well as the visual and verbal memory sub-tests in both experimental and control groups, were all within this range, suggesting normal distribution. To further verify normality, the Shapiro–Wilk test was also applied, and the results (provided in Table 3) supported the assumption of normality across all variables. Based on these normality assumptions, an independent samples *t*-test was used to compare the experimental and control groups, while a paired samples *t*-test was utilized to examine within-group differences over time. To account for the increased risk of Type I error due to multiple comparisons, Bonferroni correction was applied. Given that eight t-tests were conducted (four within-group and four between-group comparisons), the adjusted alpha level was set to *p* < 0.006. This adjustment ensured the robustness of the statistical analyses.

Cohen’s d was calculated as a measure of effect size, evaluating both the power and significance of the findings. The effect size, determined by dividing the mean difference between groups by the pooled standard deviation, is categorized into small (0.20), medium (0.50), and large (0.80) effects, as per Cohen’s (1988) guidelines. The calculated effect sizes are discussed in light of their implications for the study’s impact and alignment with the previous literature on WM interventions. All sessions were recorded online and later reviewed by an independent observer using a structured checklist to assess intervention fidelity.

To ensure implementation reliability, the Working Memory Intervention Program Session Checklist was developed, and each session’s adherence to this checklist was evaluated. These checklists included specific instructional components, such as session routines, strategy teaching, and feedback mechanisms. Key components of the checklist included the following:

- Providing students with information about the session’s purpose and strategies;
- Explaining and modeling target strategies;
- Allowing students to practice strategies through activities;
- Summarizing key points while providing constructive feedback.

All intervention sessions were conducted via Zoom and recorded for later analysis. A special education expert independently reviewed 25% of these recorded sessions using the control lists. The evaluation confirmed a fidelity rate of 96.25%, indicating a high level of adherence to the planned instructional components. This high level of consistency demonstrates that the intervention was delivered as intended, thereby enhancing the reliability of the study’s findings.

## 3. Results

This study utilized an experimental design to examine the effects of a WM intervention package, developed to improve WM performance, on the WM performance of students with SLD.

**Figure A1:**
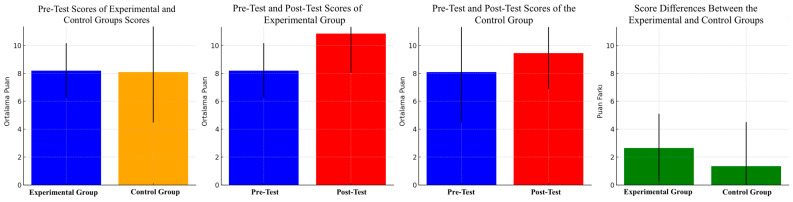
Visual Memory Scores Pre-Test and Post-Test Data for the Experimental and Control Groups.

Findings of the Visual Memory Subtest: The experimental group’s visual memory scores increased significantly from a pre-test mean of *M* = 8.20 (*SD* = 1.96) to a post-test mean of *M* = 10.85 (*SD* = 2.81), with a large effect size (Cohen’s *d* = 1.09, *p* < 0.006). In contrast, the control group’s scores showed a small and non-significant increase from *M* = 8.50 (*SD* = 3.09) to *M* = 9.45 (*SD* = 2.56, *p* = 0.09). These findings demonstrate the intervention’s strong impact on the experimental group’s visual memory performance, while natural development in the control group appears limited and statistically insignificant. Detailed results are presented in Table 4, and visual memory findings are illustrated in Figure A1.

**Figure A2:**
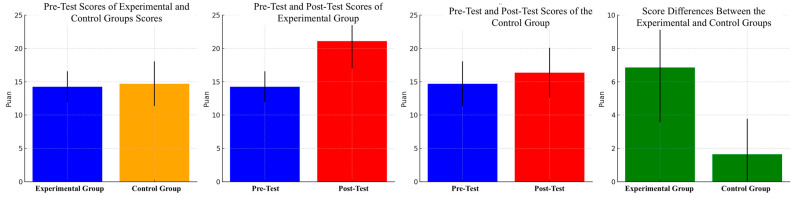
Verbal Memory Scores Pre-Test and Post-Test Data for the Experimental and Control Groups.

Findings of the Verbal Memory Subtest: The experimental group’s verbal memory scores increased significantly from a pre-test mean of *M* = 14.25 (*SD* = 2.31) to a post-test mean of M = 21.10 (SD = 4.12), with a large effect size (Cohen’s *d* = 2.09, *p* < 0.006). In contrast, the control group’s scores showed a statistically significant improvement from *M* = 14.70 (*SD* = 3.35) to *M* = 16.35 (*SD* = 3.74), with a moderate effect size (Cohen’s *d* = 0.77, *p* = 0.002). These findings demonstrate the intervention’s strong impact on the experimental group’s verbal memory performance, while the control group exhibited statistically significant but smaller improvements. Detailed results are presented in Table 4, and verbal memory findings are illustrated in Figure A2.

**Figure A3:**
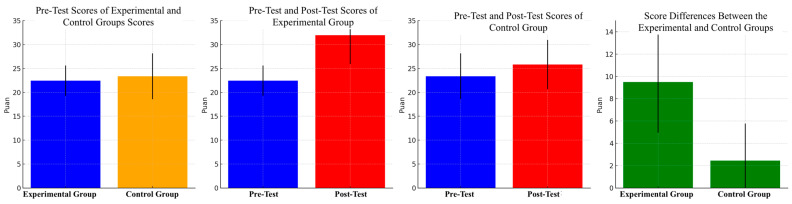
WMS Scores Pre-Test and Post-Test Data for the Experimental and Control Groups.

Findings Related to the Working Memory Scale: The experimental group’s WMS scores increased significantly from a pre-test mean of *M* = 22.45 (*SD* = 3.17) to a post-test mean of *M* = 31.95 (*SD* = 6.07), with a large effect size (Cohen’s *d* = 1.96, *p* < 0.006). The control group also showed a statistically significant improvement from M = 23.35 (SD = 4.80) to *M* = 25.80 (*SD* = 5.14), but with a moderate effect size (Cohen’s *d* = 0.49, *p* < 0.006). The experimental group’s average increase (9.5 points) was significantly greater than the control group’s (2.45 points), as confirmed by a statistically significant difference (*t*(38) = 5.45, *p* < 0.006), highlighting the intervention’s stronger impact on the experimental group. Detailed results are presented in Table 4, and WMS findings are illustrated in Figure A3.

**Figure A4:**
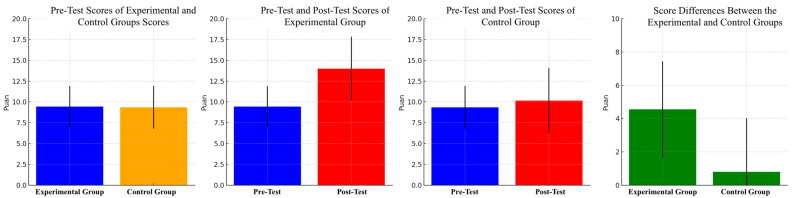
WMS-PTF Scores Pre-Test and Post-Test Data for the Experimental and Control Groups.

Findings of the Working Memory Performance Tasks Form Scores: The experimental group’s WM-PTF scores increased significantly from a pre-test mean of *M* = 9.45 (*SD* = 2.44) to a post-test mean of *M* = 14.00 (*SD* = 3.83), with a large effect size (Cohen’s *d* = 1.42, *p* < 0.006). In contrast, the control group’s scores showed a modest and non-significant increase from *M* = 9.35 (*SD* = 2.56) to *M* = 10.15 (*SD* = 3.90), with a small effect size (Cohen’s *d* = 0.24; *p* = 0.28). The experimental group’s average increase (4.55 points) was significantly greater than the control group’s (0.80 points), as confirmed by a statistically significant difference, (*t*(38) = 3.15, *p* = 0.003), favoring the experimental group. Detailed results are presented in Table 4, and WM-PTF findings are illustrated in Figure A4.

Findings on the Relationship Between the Experimental Group’s Working Memory Scale Score Changes and Intelligence Test Scores: This study examined the association between changes in WMS scores and the intelligence test results of students in the experimental group using correlation and regression analyses. The score differences between the pre-test and post-test were compared to their intelligence test outcomes to assess the strength of the relationship between these variables. The findings are displayed in Table 5.

The correlation analysis revealed a moderate positive relationship between the WMS score change and intelligence test scores in the experimental group (*r* = 0.53). This relationship indicates that intelligence test scores explain approximately 28.3% of the variance in WMS score changes (*R*^2^ = 0.28). The correlation was statistically significant (*p* = 0.01), suggesting a notable relationship between intelligence test scores and changes in WMS scores. In the control group, the correlation analysis revealed a low positive relationship between the change in WMS scores and intelligence test scores (*r* = 0.23). This indicates that intelligence test scores explain only about 5% of the variance in WM score changes (*R*^2^ = 0.05). The correlation was not statistically significant (*p* = 0.31). These findings suggest that the association between intelligence scores and changes in the WMS score was weaker and not meaningful in the absence of targeted interventions.

**Figure A5:**
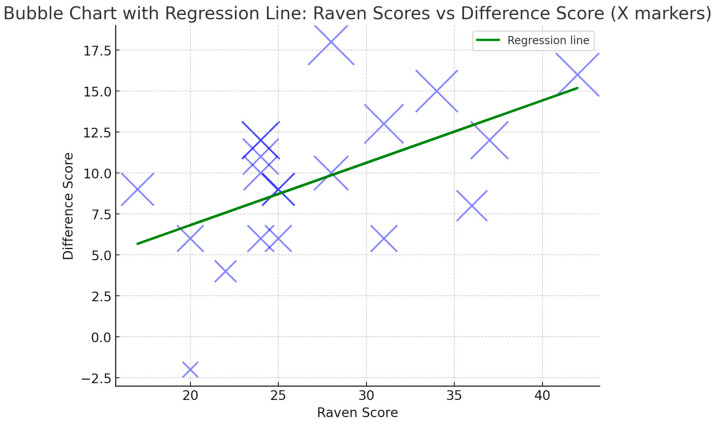
Raven Scores and Score Change in the WMS Score (X markers). Note: The X symbol represents the change in the WMS score.

To examine whether the relationship between intelligence test scores and WMS score changes differed between experimental and control groups, a moderation analysis was conducted. The moderation analysis revealed a significant interaction effect (*b*_3_ = 0.234, *p* < 0.05), indicating that the relationship between intelligence test score and WMS score changes differed between experimental and control groups. Specifically, the relationship was stronger in the experimental group. The main effects of intelligence (*b*_1_ = 0.525, *p* < 0.05) and group (*b*_2_ = 5.320, *p* < 0.01) were also significant, with higher intelligence scores and experimental group membership being associated with greater score changes. Additionally, the correlation and regression findings are illustrated in Figure A5.

## 4. Discussion

This study aims to assess the impact of a WM intervention package on the cognitive performance of students with SLD, comparing outcomes between the experimental and control groups. The intervention, designed to enhance students’ memory and recall abilities, emphasizes cognitive and metacognitive strategies. While the existing literature primarily examines overall WM performance, the specific relationship between WM and SLD remains underexplored (Swanson and Siegel 2011). This study emphasizes the need for tailored interventions targeting specific cognitive profiles and highlights the importance of further research into the connections between SLD and WM components to inform effective intervention strategies. This study’s findings align with the literature, demonstrating the positive effects of WM intervention training on WM performance. The experimental group showed significant improvements in WM capacity compared to the control group, which exhibited smaller but meaningful gains. Targeted WM interventions have been shown to significantly improve WM capacity and recall processes, as well as attention performance, particularly in students with ADHD and SLD (Klingberg et al. 2005; Witt 2011; Hovik et al. 2013; Wiest et al. 2022). Recent meta-analyses (Peijnenborgh et al. 2016; Rao et al. 2023) further highlight statistically significant enhancements in verbal and visual WM compared to control conditions, supporting their efficacy in educational settings for students with SLD. Systematic reviews and meta-analyses support the effectiveness of WM interventions in enhancing the WM capacities of individuals with cognitive limitations, including those with SLD, ADHD, and intellectual disabilities (Alaqeel and Aldoghmy 2018). Wiest et al. (2022) demonstrated that memory-based cognitive training not only improved WM performance but also enhanced attentional capacities, particularly in students with SLD and ADHD. Peijnenborgh et al. (2016) and Rao et al. (2023) highlighted the significant impact of well-structured and tailored interventions, documenting substantial improvements in verbal and visual WM among students with SLD. These findings emphasize the importance of precision-targeted approaches and sustained effort to achieve lasting cognitive gains. Additionally, Dehn (2008) advocates for early identification of cognitive deficits in SLD populations to optimize intervention outcomes, while Redick (2019) observed that WM training can transfer positively to related cognitive skills beyond the targeted tasks. These results align with existing research, highlighting the positive effects of WM interventions on memory and their potential as effective educational tools for students with SLD.

Discussion of Visual Memory: This study explores why the observed increase in visual memory performance in the experimental group did not result in a statistically significant difference compared to the control group. The experimental group exhibited significant improvements in visual memory scores, reflecting the positive impact of the intervention. However, this improvement did not translate into a meaningful difference between groups, potentially due to various external and individual factors.

Individuals with dyslexia often demonstrate stronger visual processing and recall abilities, as noted in prior studies (Von Karolyi 2001; Pillai and Yathiraj 2017). This natural inclination toward visual information processing may have contributed to the observed improvements in the experimental group. Furthermore, auditory processing difficulties, such as phonological deficits, are commonly cited as barriers to encoding and recalling auditory information in individuals with dyslexia and SLD (Shaywitz 2003). In this context, the improvements in visual memory performance may reflect a preference for visual information processing in students with SLD (Riddick 2001).

Research by Van Dongen-Boomsma et al. (2014) and Roberts et al. (2016) demonstrated improvements in verbal WM tasks but found limited gains in visual memory. These findings suggest that visual memory may require more tailored approaches to achieve significant improvements. Technology-based and interactive interventions have been noted to have a stronger impact on visual memory (Chen et al. 2018). However, this study adopted a group training approach without implementing individualized educational strategies, which may explain the limited progress observed.

Several factors may account for the lack of a statistically significant difference between the experimental and control groups. First, the repeated test administration likely increased students’ familiarity with the questions, positively influencing performance through the testing effect (Karpicke and Roediger 2008). Second, students in the control group may have had incidental exposure to visual-memory-enhancing materials at the rehabilitation centers they attended. Finally, students with SLD often display variable performance across different times, which may have influenced the results (Smith and Strick 2010). Although the experimental group demonstrated significant improvements, the findings align with prior research suggesting that interventions focusing on auditory or verbal memory yield more pronounced results (Van Dongen-Boomsma et al. 2014; Roberts et al. 2016). This study highlights the importance of tailoring interventions to address the specific needs of students with SLD. The inclusion of technology-based methods, as Chen et al. (2018) suggested, may further enhance outcomes by leveraging interactive tools to support visual memory improvements.

In conclusion, while significant improvements in visual memory were observed in the experimental group, the lack of a statistically significant difference compared to the control group may be attributed to intervention methods, testing effects, and individual differences among participants. These findings underscore the importance of designing educational interventions and assessment tools that consider individual characteristics and learning profiles.

Discussion of Verbal Memory: In cases of SLD and dyslexia, verbal memory performance is a primary area requiring intervention (Swanson and Howell 2001; Ramus 2003). In this study, the most significant improvement was observed in verbal memory performance, emphasizing the positive impact of targeted strategies, such as rehearsal and chunking (Turley-Ames and Whitfield 2003). Enhancing verbal memory is critical for sustaining attention and processing information effectively, particularly in individuals with SLD (Gathercole and Baddeley 2014). Although the control group also showed a statistically significant improvement, these modest gains could be attributed to natural developmental processes or incidental exposure to educational activities. In contrast, the larger gains observed in the experimental group underscore the critical role of structured interventions in fostering meaningful progress. The intervention utilized strategies, such as rehearsal, chunking, and visualization, that allowed students to repeatedly practice encoding and retrieval processes, organize information into meaningful groups, and strengthen their ability to recall verbal information. Rehearsal strengthens encoding through repetition, helping students with SLD who often face challenges with retention (Turley-Ames and Whitfield 2003). Chunking facilitates the organization of information into smaller, manageable units, enhancing retrieval processes, while visualization further aids comprehension and recall by creating meaningful associations (Gathercole and Baddeley 2014). These strategies, implemented in combination, contributed significantly to the large effect size observed in the experimental group.

These findings align with previous research highlighting the positive impact of strategy instruction on verbal memory. For instance, Peng and Fuchs (2016) reported significant improvements in verbal memory among students at risk for SLD using similar approaches. Verbal working memory training has been shown to significantly enhance reading and language skills (Melby-Lervåg and Hulme 2013; Gathercole et al. 2006). Additionally, interventions targeting auditory memory improve phonological processing and reading abilities, which are critical for students with SLD (Holmes et al. 2009; Snowling 2001). As many WM interventions lack specific strategy instruction in verbal memory (Peng and Fuchs 2016), this study highlights the importance of addressing this gap. By integrating targeted strategies into intervention programs, the unique learning profiles of students with SLD can be addressed, significantly enhancing verbal memory and broader cognitive and academic performance (Highnam and Sheppard 1992; Dehn 2015). Tailoring interventions to individual profiles not only optimizes outcomes for students with learning difficulties but also supports their overall cognitive development.

In conclusion, this study demonstrates the effectiveness of rehearsal, chunking, and visualization techniques in enhancing verbal memory performance. The observed improvements highlight the potential of structured and targeted WM interventions to address the cognitive needs of students with SLD. By tailoring these strategies to individual profiles, such interventions offer considerable promise for improving learning outcomes and supporting academic achievement (Highnam and Sheppard 1992; Dehn 2015).

Discussion of WM-PTF: This study highlights that WM-PTF tasks are designed to evaluate individuals’ ability to temporarily store and process information, testing WM capacity (Cowan 2008). Traditional WM tasks, such as auditory and visual digit spans and the N-Back task, are widely used in education, clinical assessment, and cognitive research (Conway et al. 2005; Jaeggi et al. 2008). In this study, the experimental group demonstrated a statistically significant improvement in WM-PTF scores, with a large effect size. This improvement reflects a strong enhancement of their ability to handle cognitive load. In contrast, the control group showed a smaller and statistically non-significant increase in WM-PTF scores. The significant difference between the experimental and control groups highlights the stronger impact of the targeted intervention in improving WM capacity. The increase in WM-PTF scores suggests that students’ processing capacity in WM tasks has improved, reflecting an enhancement in their ability to handle cognitive load, the mental effort required to process information during learning (Paas and Van Merrienboer 2020). Cognitive load theory posits that working memory has a limited capacity, which can be overwhelmed by complex information, impeding learning and retention. This capacity can be optimized through strategies like chunking information and simplifying learning materials, which reduce unnecessary cognitive load and promote more efficient processing (Paas et al. 2003; Mayer and Moreno 2003). These approaches help structure learning processes and enhance learning efficiency (Paas and Van Merrienboer 2020). The significant improvement observed in the experimental group suggests that the intervention not only helped optimize cognitive load but also enabled students to manage complex learning tasks more effectively. By targeting the WM-PTF specifically, this intervention addressed students’ ability to process and store information simultaneously, which is a critical skill in learning environments (Sweller et al. 2011). Research on students with SLD highlights the essential role of WM tasks in improving cognitive performance and addressing learning difficulties (Greenberg and Zheng 2022). Proper strategy training has been shown to significantly enhance learning performance, while WM training improves attention, problem solving, and general cognitive functions (Klingberg et al. 2002; Jaeggi et al. 2008). The findings of this study align with prior research demonstrating that WM interventions enhance students’ ability to process and store information more efficiently (Novaes et al. 2019; Peng and Fuchs 2016). These interventions have also been shown to improve cognitive functions, such as attention and problem solving, by reducing cognitive overload and increasing processing efficiency (Sweller et al. 2011). The effectiveness of WM interventions depends on tailoring them to the individual differences among students. For students with SLD, these interventions enable them to manage cognitive load and process learning materials more efficiently, which is essential for improving academic performance (Rao et al. 2023). These findings underscore the critical role of WM tasks and cognitive strategy training in facilitating the learning process.

Discussion of the Relationship Between Changes in WMS Performance and Intelligence: This study examined the relationship between changes in WMS scores and intelligence scores, revealing a moderate correlation in the experimental group. These findings support the connection between WM capacity and higher-order cognitive functions, such as intelligence, as suggested by prior research (Dehn 2015; Klingberg 2010). The results also highlight the role of individual differences, particularly intelligence levels, in shaping the outcomes of cognitive interventions. Participants with higher intelligence scores demonstrated greater improvements in WM performance, consistent with the findings of Sternberg (1997), Veenman and Spaans (2005), and Alexander et al. (1995). Higher intelligence enhances the ability to learn and apply cognitive and metacognitive strategies, which in turn supports WM development. For instance, higher intelligence may facilitate the acquisition and application of organizational strategies during WM tasks, resulting in more pronounced performance improvements (Sternberg 1997; Veenman and Spaans 2005). The stronger correlation observed in the experimental group suggests that targeted interventions may maximize the relationship between intelligence and WM changes. In contrast, the control group exhibited a weaker and statistically non-significant correlation, indicating that without structured interventions, natural changes in WM performance are not strongly associated with intelligence levels.

To explain the relationship between WMS score changes and intelligence, several theoretical frameworks are proposed. First, the cognitive efficiency hypothesis, as suggested by Barbey et al. (2014) and Kane and Engle (2002), posits that individuals with higher intelligence allocate cognitive resources more efficiently during WM tasks. This efficiency may account for why intelligence serves as a stronger predictor of WM improvements in the experimental group. Second, the strategy use and metacognition model, supported by Sternberg (1997) and Veenman and Spaans (2005), emphasizes the role of intelligence in metacognitive processes, where higher intelligence enables the development and application of effective encoding and retrieval strategies during WM tasks (Alexander et al. 1995; Dehn 2015). In the control group, the correlation between changes in WM performance and intelligence was found to be low (*r* = 0.23) and statistically non-significant (*p* = 0.31). This finding indicates that in the absence of targeted interventions, there is no significant relationship between changes in WM performance and intelligence. The findings emphasize the importance of targeted interventions in strengthening the relationship between WM and intelligence. While intelligence test scores explained 28.3% of the variance in WM changes in the experimental group, a substantial portion of the variance remains unexplained. This highlights the need to explore additional factors contributing to WM improvements, such as motivation, baseline WM capacity, or environmental influences.

Moreover, the observation that the relationship between WM changes and intelligence weakens as intelligence levels decrease underscores the need for interventions tailored to individuals with lower cognitive capacities. Adaptive training programs that adjust task difficulty based on individual performance could help bridge this gap, ensuring that participants across a broader range of cognitive abilities benefit from WM interventions (Sultanova 2024). Finally, larger sample sizes and more refined measurement tools are recommended to validate these findings and further clarify the relationship between WM changes and intelligence in non-intervention contexts. Long-term studies are also necessary to examine whether the effects of WM interventions persist over time and contribute to sustained improvements in intelligence-related abilities.

While the findings of this study provide important insights, some limitations should be noted. The study included 20 students in the experimental group and another 20 students in the control group over five weeks, consisting of 45 sessions, and was implemented as online group training. However, the limited sample size may restrict the generalizability of the findings and should be considered when interpreting the results. In the online environment, mental tasks were used instead of written tasks, which may have resulted in a loss of precision in the evaluation process. Specifically, students not receiving points due to errors made in the final steps of questions was identified as a limitation in the scoring process. Additionally, the possibility that students continued receiving special education during the summer, which could have indirectly influenced their cognitive skills and memory, should be considered. Given these factors, the results should be interpreted with caution. We recommend that future research address these limitations by utilizing more individualized interventions and a broader range of cognitive assessment tools.

## 5. Conclusions and Recommendations

### 5.1. Conclusions

This study tested five main hypothesis. The first hypothesis proposed that the WM intervention package would significantly improve visual memory performance in the experimental group compared to the control group. Although the results indicated improvements in visual memory within the experimental group, this improvement did not lead to a statistically significant difference when compared to the control group. This suggests that while visual memory may benefit from WM interventions, further investigation with a larger sample size may be necessary to determine a more definitive effect. The second hypothesis predicted that the WM intervention would result in greater improvements in verbal memory performance in the experimental group than in the control group. As hypothesized, participants in the experimental group demonstrated significantly greater improvements in verbal memory, indicating that the intervention had a positive effect specifically on verbal memory capabilities. The third hypothesis posited that the WM intervention would lead to statistically significant gains in overall WMS scores in the experimental group relative to the control group. The findings supported this hypothesis, revealing that the experimental group showed a significant improvement over the control group, with a larger effect size observed in the experimental group, underscoring the intervention’s effectiveness in enhancing broader WM skills. The fourth hypothesis suggested that the WM intervention would significantly enhance WM-PTF performance in the experimental group compared to the control group. The results supported this hypothesis, as the experimental group experienced significantly greater improvement in WM-PTF tasks than the control group, indicating that the intervention effectively enhanced participants’ ability to process and store information. The fifth hypothesis explored whether changes in WMS scores would positively correlate with intelligence scores. The results confirmed this hypothesis, identifying a moderate positive correlation between WM improvements and intelligence scores. These findings suggest that intelligence scores may play a role in the degree of improvement observed in WM performance, with higher intelligence scores associated with greater gains.

In summary, the results of this study demonstrate that the WM intervention package was effective in enhancing various aspects of WM performance, particularly in students with SLD, with notable improvements in verbal memory, overall WMS scores, and WM-PTF tasks.

### 5.2. Recommendations

Practical Recommendations: Cognitive intervention programs for students with SLD can be expanded. The effects of such interventions on other cognitive skills, such as attention and executive functions, can be examined. The effectiveness of different intervention content can be compared. Intervention programs targeting both visual and verbal memory can be developed.

Recommendations for Future Research: WM training can be implemented in a more individualized manner, and its long-term effects can be monitored. The impact of WM training on different diagnostic groups and settings can be explored. The relationship between WM, ADHD, and academic skills can be examined in detail. These recommendations will guide the development of WM interventions and future research, contributing significantly to the practical application of WM interventions and informing future studies.

## Figures and Tables

**Table 1:** Demographic and descriptive features of the participants.

Descriptive Features of Groups		Experimental Groups		Control Groups		Total	
		*n* %		*n* %		*n* %	
Gender	Female	8	40%	10	50%	18	45%
	Male	12	60%	10	50%	22	55%
Grade Level	3rd grade	9	45%	5	25%	14	35%
	4th grade	11	55%	15	75%	26	65%
Total		20	50%	20	50%	40	100%
Mean Age and Test Scores		M	SS	M	SS	Total	
Mean Age		9.505	0.35	9.81	0.511	9.71	
RSPM Test Mean		27.05	6.22	26.6	4.59	26.82	
IQ Score		100.68	15.89	99.21	14.19	99.94	
WMS Pre-Test Mean		22.45	3.17	23.35	4.80	22.9	

WMS: Working Memory Scale; WM-PTF: Working Memory–Performance Task Form; RSPM: Raven’s Standard Progressive Matrices; IQ: intelligence quotient.

**Table 2:** Objectives of the Working Memory Intervention Package and student outcomes.

Weeks	Sessions	Objectives	Student Outcomes
1	9	Introduction activities with students Key aspects of online learning What are memory and WM? What are their characteristics? Demonstration of practice examples	Students meet their group peers. They gain basic knowledge about the intervention process and its applications. They learn to use online education software. They understand the fundamental characteristics of WM and grasp practice examples related to WM.
2	9	Strategy instruction through practice examples Rehearsal Chunking Visualization	Students learn strategy instruction through direct teaching via practice examples. They develop the ability to independently apply their learned strategies and begin practicing them.
3	9	Phonological STM Visual–Spatial STM Phonological + Visual STM	Students develop the capacity to implement the strategies they have acquired and begin performing the activities at the required level.
4	9	Phonological + Visual–Spatial STM Verbal WM Visual–Spatial WM	Students begin performing the activities at the desired level by applying the strategies they have learned, and they are expected to develop the capability to manage the process fluently.
5	9	Verbal WM Visual–Spatial WM Executive WM	Students independently select the most appropriate strategy from the available options and manage the process, developing the ability to perform activities based on their chosen strategy.

WM: working memory; STM: short-term memory.

**Table 3:** Skewness and kurtosis statistics for the pre-test and post-test scores in the study.

Groups	Variable Names	WMS Pre-Test	WMS Post-Test	WM-PTF Pre-Test	WM-PTF Post-Test	Verbal Memory Scores Pre-Test	Verbal Memory Scores Post-Test	Visual Memory Scores Pre-Test	Visual Memory Scores Sub-Test
Exp.	Kurtosis	−0.32	1.84	−0.37	−0.99	0.37	0.60	−1.23	−0.23
	Skewness	0.19	−1.11	0.24	−0.06	0.42	−0.78	−0.28	−0.43
Control	Kurtosis	−1.16	−0.30	0.70	−1.38	−0.83	−0.59	0.49	1.50
	Skewness	0.003	0.19	1.12	0.29	−0.16	0.34	0.29	−1.13
Total	Kurtosis	−0.73	−0.41	0.18	−1.07	−0.38	−0.73	0.90	0.65
	Skewness	0.17	−0.23	0.71	0.06	0.06	−0.07	0.26	−0.60

Exp: experimental; WMS: Working Memory Scale; WM-PTF: Working Memory–Performance Task Form.

**Table 4:** Findings of WM and subtest scores for the experimental and control groups.

Measurement Area	Group	Pre-Test (M)	SS	Post-Test (M)	SS	Paired *t*-Test	Within-Group Difference (*p*)	Between-Group Difference (*p*) (t)	Effect Size (*d*)
Visual Memory Scores	Exp. * (*n* = 20)	8.20	1.96	10.85	2.81	−4.83	*p* = 0.000 *p* < 0.006	*p* = 0.10 *p* > 0.006 t = 1.64	*d* = 1.09
	Control (*n* = 20)	8.50	3.09	9.45	2.56	−1.76	*p* = 0.09 *p* > 0.006		*d* = 0.33
Verbal Memory Scores	Exp. * (*n* = 20)	14.25	2.31	21.10	4.12	−9.33	*p* = 0.000 *p* < 0.006	*p* = 0.000 *p* < 0.006 t = 3.81	*d* = 2.09
	Control (*n* = 20)	14.70	3.35	16.35	3.74	−3.45	*p* = 0.002 *p* < 0.006		*d* = 0.77
WMS	Exp. * (*n* = 20)	22.45	3.17	31.95	6.07	−9.34	*p* = 0.000 *p* < 0.006	*p* = 0.001 *p* < 0.006 t = 3.45	*d* = 1.96
	Control (*n* = 20)	23.35	4.80	25.80	5.14	−3.30	*p* = 0.004 *p* < 0.006		*d* = 0.49
WM-PTF	Exp. * (*n* = 20)	9.45	2.44	14.00	3.83	−7.04	*p* = 0.000 *p* < 0.006	*p* = 0.003 *p* < 0.006 t = 3.15	*d* = 1.42
	Control (*n* = 20)	9.35	2.56	10.15	3.90	−1.11	*p* = 0.28 *p* > 0.006		*d* = −0.24

Note: Bonferroni correction was applied to control for multiple comparisons, with the adjusted alpha level set to *p* < 0.006. Tests with *p*-values below this threshold were considered statistically significant. * Exp: experimental; WMS: Working Memory Scale; WM-PTF: Working Memory–Performance Task Form.

**Table 5:** Correlation table between the experimental and control group’s Working Memory Scale score changes and intelligence test scores.

		N	M	ss	D	R^2^	*p*
Correlation Between the Experimental Group’s Working Memory Scale Score Changes and Intelligence Test Scores	RSPM Test Score	20	27.05	6.36	0.53	0.28	0.01
	Score Change in WMS Test	20	9.50	4.55			
Correlation Between the Control Group’s Working Memory Scale Score Changes and Intelligence Test Scores	RSPM Test Score	20	26.60	4.59	0.23	0.05	0.31
	Score Change in WMS Test	20	2.50	3.32			

WMS: Working Memory Scale; RSPM: Raven’s Standard Progressive Matrices.

## Data Availability

The original data presented in the study are openly available in FigShare at https://doi.org/10.6084/m9.figshare.27141603 (accessed on 22 January 2025).

## Appendix A

This appendix contains detailed visualizations that illustrate the pre-test and post-test score averages, along with the change scores for the experimental and control groups. These visualizations cover key cognitive areas, specifically visual memory, verbal memory, WMS scores, and WM-PTF scores.
